# Pyogenic vertebral osteomyelitis complicating abdominal penetrating injury: case report and review of the literature

**DOI:** 10.1186/1749-7922-8-56

**Published:** 2013-12-27

**Authors:** Arianna Zefelippo, Paola M Bertazzoni, Aldo M Marini, Paolo De Rai, Ettore Contessini-Avesani

**Affiliations:** 1 General and Emergency Surgery Unit, Fondazione IRCCS Cà Granda Ospedale Maggiore Policlinico di Milano, via F. Sforza 35-20100, Milano, Italy; 2 General Surgery Post-graduation School, University of Milan, via F. Sforza 35-20100, Milano, Italy

**Keywords:** Vertebral osteomyelitis, Penetrating abdominal trauma, Post-traumatic infections

## Abstract

Pyogenic vertebral osteomyelitis is a rare condition usually associated with endocarditis or spinal surgery. However, it may also occur following abdominal penetrating trauma with associated gastrointestinal perforation. Diagnosis might be challenging and appropriate treatment is essential to ensure a positive outcome. In trans-abdominal trauma, 48 hours of broad-spectrum antibiotics is generally recommended for prophylaxis of secondary infections. A case report of vertebral osteomyelitis complicating trans-colonic injury to the retroperitoneum is presented and clinical management is discussed in the light of literature review.

## Background

Pyogenic vertebral osteomyelitis is a rare condition usually related to endocarditis or spinal procedures [1,2]. It may also develop as a complication of penetrating trauma of the spine [3] and it has been reported in literature in association with abdominal low-speed gunshot wounds [4-7] or stab wounds [8,9]. Trans-colonic injuries in particular appear to be at higher risk of developing secondary infections [3,10]. Diagnosis of vertebral osteomyelitis might be challenging due to subtle onset of symptoms and unspecific clinical features. Persistent back pain and fever, sometimes associated with neurological impairment, are the usual findings [1]. However, in trauma patients concurrent injuries may masquerade symptoms and delay diagnosis. Etiological diagnosis and correct clinical management are essential to ensure an appropriate therapy and to avoid complications. Treatment usually requires a long course of antibiotics and prolonged bed rest [2]. A case report of vertebral osteomyelitis complicating trans-colonic injury to the retroperitoneum is presented alongside a review of the literature.

### Case presentation

**Figure 1:**
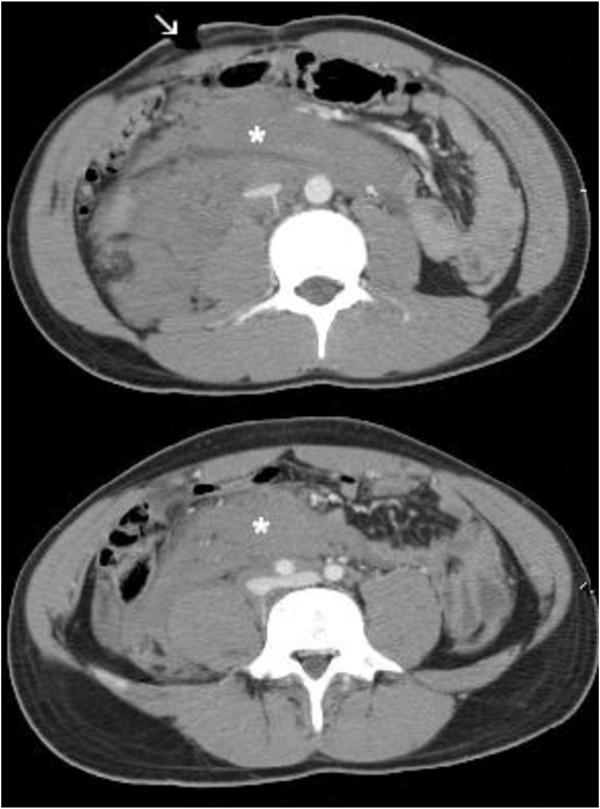
**CT scan on admission.** CT scan on admission showed a large retroperitoneal hematoma (*). Entrance site of penetrating wound is visible at right lower quadrant (arrow).

**Figure 2:**
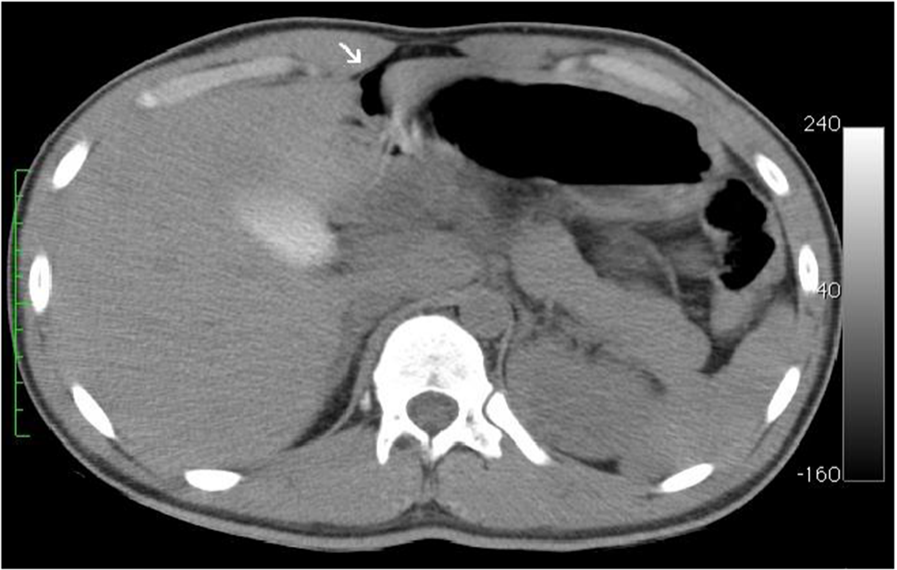
**Repeated CT scan.** A CT scan was repeated after the patient developed peritonitis. Peritoneal free air was detected (arrow).

A 21 year-old male was admitted to the emergency department for abdominal penetrating injury by a pointed metal stick (namely, a doner kebap spit). On primary survey, vital signs were normal and clinical examination demonstrated a single penetrating wound at the right inferior abdominal quadrant. No peritoneal free fluid was detected on ultrasound scan. Tetanus prophylaxis was administered. A thoraco-abdominal computed-tomography (CT) scan showed a retroperitoneal hematoma surrounding the sub-hepatic inferior vena cava with no intraperitoneal fluid or other abnormalities (Figure 1). A minimal tear of the vena cava was suspected to be the source of bleeding; due to hemodynamic stability, the patient was initially treated conservatively. After three hours of clinical observation, he developed peritonitis while vital signs remained normal and steady. Thoraco-abdominal CT scan was repeated in order to rule out any rebleeding in the retroperitoneum and to investigate possibility for endovascular treatment prior to surgery. The hematoma was unchanged compared to the first scan whereas free peritoneal air was demonstrated (Figure 2). At laparotomy, diffuse peritonitis secondary to perforation of the transverse colon was found. Perforation was repaired with direct suture and a sample of peritoneal fluid was collected for cultures. Retroperitoneum was left untouched. Postoperative recovery was uneventful. The patient received 5 days of intravenous broad spectrum antibiotics (imipenem) and was discharged in 8 days.

**Figure 3:**
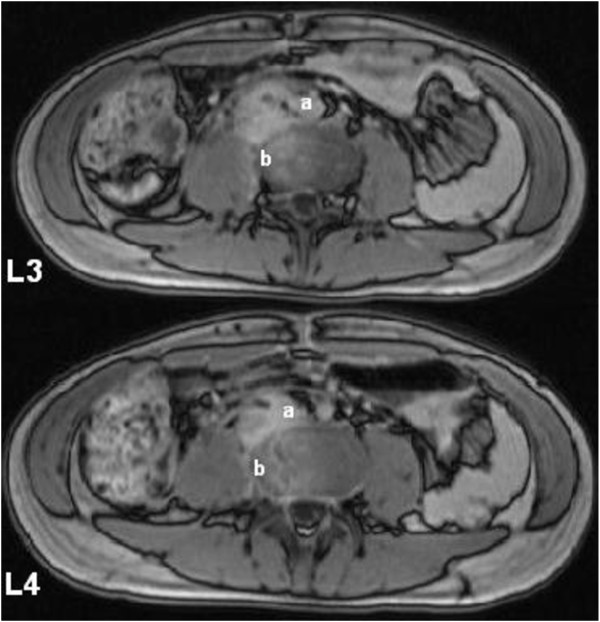
**Diagnostic MRI.** Contrast MRI demonstrated a small paravertebral collection **(a)** and osteomyelitis at L III – L IV with areas of bone erosion **(b)** (T1 weighted images are shown).

Ten days later he was readmitted for fever and worsening lumbar pain radiating to the limbs bilaterally with minimal walking impairment. Blood exams revealed increased C-reactive protein (CRP) levels and leukocyte count. Abdominal CT scan showed no signs of intra or retroperitoneal abscess; the retroperitoneal hematoma appeared decreased in size. No evidence of chest or urinary tract infections was demonstrated. Eventually, magnetic resonance imaging (MRI) showed osteomyelitis at III and IV lumbar vertebrae with bone erosion and inflammation of disc space; a small collection in the paravertebral tissue at that level was also detected (Figure 3). No vertebral fractures or spinal involvement were demonstrated and clinical assessment was performed to confirm spinal stability. Given the results of cultures on peritoneal fluid collected at time of laparotomy, that showed polimicrobial contamination by Escherichia coli, Enterobacter cloacae, Candida albicans and Candida krusei, treatment was started with intravenous piperacillin/tazobactam and fluconazole. Ten sessions of hyperbaric oxygen therapy (HBOT) were administered in addition. Analgesia and bed rest were effective in alleviating symptoms. Clinical response to therapy was satisfactory and CRP levels were decreased after 2 weeks of treatment. Repeated sets of blood cultures were negative. The patient was discharged in 20 days on oral medications (ciprofloxacin, thrimethoprim and fluconazole) for 6 weeks and prescription for a back brace and physiotherapy. Clinical improvement was confirmed at 10 days follow-up. He made a full recovery in 2 months.

## Discussion

Pyogenic vertebral osteomyelitis is a rare disease that counts for 2-5% of all cases of osteomyelitis, with an annual incidence of 0.4 to 2.4/100′000 among European population [2]. Predisposing factors are intravenous drug use, immunosuppression, chronic illnesses and insulin-dependent diabetes mellitus. Typically, vertebral osteomyelitis is a complication of bacterial endocarditis and septicemia. Direct contamination associated with spinal surgery or epidural procedures appears to be of increasing importance among possible etiologies [1,2]. According to observational studies, Staphylococcus aureus (20-84%) and Enterobactericeae (33%) are the most common pathogens, with anaerobes (3%) and fungi (1-2%) rarely involved; less than 10% are polimicrobial infections [11]. In trauma setting, direct or trans-abdominal penetrating injuries to the spine are at risk of developing secondary infections, particularly when a hollow viscus is perforated [3,10]. In the presented case, a pointed metal stick caused a perforation of the transverse colon and a retroperitoneal injury. Bone infection was considered to be secondary to direct contamination from the peritoneum and treated accordingly. Diagnosis of pyogenic vertebral osteomyelitis is usually guided by clinical suspicion in the presence of persistent back pain and remitting fever. A wide range of neurological symptoms, including weakness, radiculopathy and sensory loss may also be shown by up to 30% of the patients [1]. It has to be noted that in trauma patients, concurrent injuries may mislead and delay diagnosis. In our case, fever, back pain and neurological impairment were at first attributed to superinfection of the retroperitoneal hematoma or possibly to an intra-abdominal abscess, before diagnosis of vertebral osteomyelitis was made. Adequate imaging should also support the clinical suspicion. In the presented case, CT scan of the abdomen failed to detect vertebral osteomyelitis that was subsequently diagnosed on MRI. Although plain X-ray and CT are frequently used as first step investigation for back pain, MRI is considered to be the gold standard for diagnosis of osteomyelitis. Moreover, MRI is superior to CT in defining involvement of neuronal and soft tissue and extension of the infective process [2]. Every effort should be taken to identify the pathogen, in order to ensure an appropriate antimicrobial therapy and prevent complications such as abscesses, extension of the infection to neuronal tissue, persistence or recurrence of infection, septicemia. Blood cultures have a high rate of positivity, reported to range between 30 and 75% [1]. If negative, percutaneous CT-guided biopsy to obtain material for cultures is generally recommended. Surgical biopsy in not recommended unless surgery has already been planned to drain an abscess or to treat spinal instability [2]. In our case, antimicrobial treatment was based on intraoperative cultures of peritoneal liquid whereas repeated sets of blood cultures remained negative. This therapy demonstrated to be effective and invasive diagnostic procedures were spared. 6 to 8 weeks of antibiotics is the recommended duration for treatment, which should be anyway adjusted according to clinical course. A positive response to therapy is defined by clinical improvement and decrease in CRP levels within 4 weeks [1]. Repeated MRI is usually unnecessary unless treatment failure or complications are suspected [2]. Treatment should be also focused towards alleviating symptoms, with extensive use of analgesia and bed rest. An appropriate rehabilitation plan is also advisable. HBOT has been increasingly used as adjuvant therapy for bone infections. Although lacking in high quality evidence, a number of studies have suggested HBOT to be effective in enhancing leukocyte bactericidal activity and antibiotic activity in hypoxic tissues, suppressing anaerobic pathogens, inducing angiogenesis and accelerating wound healing [12]. In our case, HBOT was administered in addition to standard treatment and proved to be beneficial.

Appropriate prophylaxis for infective complications in trauma patients has been largely investigated. Colon perforation is considered to be a risk factor for developing secondary infections and use of broad-spectrum antibiotic prophylaxis is generally recommended for at least 24–48 hours [3,10]. However, few published studies address the specific issue of spinal or paraspinal and vertebral infection following penetrating injuries associated with hollow viscus perforation. Results of a review of the literature focusing on this topic are summarized in Table 1. All case series included patients with trans-abdominal injuries to the spine caused by gunshot wounds. Reported incidence of osteomyelitis is very low, even in the presence of bowel perforation. As for prophylaxis, some authors recommend prolonged antibiotic coverage for at least 5 days [5,6,13,14] while a number of studies have showed no significant difference in the incidence of secondary infections when a short-term prophylaxis is administered [4,7,15]. It has to be noted that all these studies are characterized by the small number of patients included and by retrospective design. A recently published study included 51 patients with penetrating gunshot wounds to the spine following gastrointestinal perforation observed over 8 years of activity in a level 1 trauma center [7]. Patients received up to 48 hours perioperative antibiotic prophylaxis (35% of patients) unless damage control surgery was performed or a longer course of antibiotics was required for documented infections (65%). Only 1 patient developed a spinal infection, which occurred among the group that received prolonged antibiotic treatment. The author concluded questioning the necessity for extended prophylaxis for more than 48 hours. Although current evidence on this issue is poor, a 24–48 hours prophylaxis with large-spectrum antibiotics appears to be a reasonable approach in the event of penetrating abdominal wounds associated with hollow viscus perforation.

**Table 1:** Studies on spinal infections (including vertebral osteomyelitis) secondary to trans-abdominal injuries

**Author**	**Study design**	**Mechanism of injury**	**Patients included**	**Incidence of spinal infections**	**Antibiotic coverage**
Romanick 1985 [4]	Retrospective	Low speed gunshot wounds	20	7/8 colon perforations	At least 2 days, broad spectrum
			→12 bowel perforations:		
			→ 4 upper GI tract		
			→ 8 colon		
Roffi 1989 [5]	Retrospective	Low speed gunshot wounds	42	3/14 colon perforations	Extended course (6 to 14 days)
			→14 colon perforations		
Kihtir 1991 [15]	Retrospective	Gunshot wounds	21	0/21 patients	48 hours
			→ 5 colon perforations		
Lin 1995 [13]	Retrospective	Low speed gunshot wounds	29	0/29 patients	2 to 5 days
			→ 8 colon perforations		
Kumar 1998 [14]	Retrospective	Gunshot wounds	33	0/13 colon perforations	2 to 43 days
			→ 13 colon perforations		
Quickgley 2006 [6]	Retrospective	Low speed gunshot wounds	114	4/27 bowel perforations	5 days, broad spectrum
			→ 27 bowel perforations:	(3/15 colon perforations)	
			→ 12 upper GI tract		
			→ 15 colon		
Rabinowitz 2012 [7]	Retrospective	Gunshot wounds	51 bowel perforations:	1/51 bowel perforations	24-48 hours broad spectrum for prophylaxis *vs* prolonged treatment for documented infections
			→ 25 upper GI tract		
			→ 26 colon		

## Conclusion

Pyogenic vertebral osteomyelitis is a rare complication that may occur following abdominal penetrating injury, especially when bowel is perforated. Diagnosis can be difficult and should be guided by high clinical suspicion. An accurate management and appropriate treatment are essential to ensure a positive outcome. 48 hours of broad-spectrum antibiotics is suggested for prophylaxis of secondary infections following trans-abdominal trauma.

## Consent

Written informed consent was obtained from the patient for publication of this case report and accompanying images.

## Competing interests

The authors declare that they have no competing interests.

## Authors’ contributions

AZ was involved in the clinical management of the patient and drafted the manuscript. PD and PB performed the operation and contributed in conceiving the manuscript. AM admitted the patient and reviewed the manuscript. All authors read and approved the final manuscript.
